# Delivering the pain: an overview of the type III secretion system with special consideration for aquatic pathogens

**DOI:** 10.1186/s13567-021-01015-8

**Published:** 2021-12-19

**Authors:** Hadis Rahmatelahi, Mansour El-Matbouli, Simon Menanteau-Ledouble

**Affiliations:** 1 Clinical Division of Fish Medicine, University of Veterinary Medicine, 1210 Vienna, Austria; 2 Department of Chemistry and Bioscience, Aalborg University, Fredrik Bajers Vej 7H, 9220 Aalborg Ø, Denmark

**Keywords:** virulence, effector proteins, *Yersinia* spp., *Aeromonas* spp., *Edwardsiella* spp.

## Abstract

Gram-negative bacteria are known to subvert eukaryotic cell physiological mechanisms using a wide array of virulence factors, among which the type three-secretion system (T3SS) is often one of the most important. The T3SS constitutes a needle-like apparatus that the bacterium uses to inject a diverse set of effector proteins directly into the cytoplasm of the host cells where they can hamper the host cellular machinery for a variety of purposes. While the structure of the T3SS is somewhat conserved and well described, effector proteins are much more diverse and specific for each pathogen. The T3SS can remodel the cytoskeleton integrity to promote intracellular invasion, as well as silence specific eukaryotic cell signals, notably to hinder or elude the immune response and cause apoptosis. This is also the case in aquatic bacterial pathogens where the T3SS can often play a central role in the establishment of disease, although it remains understudied in several species of important fish pathogens, notably in *Yersinia ruckeri*. In the present review, we summarise what is known of the T3SS, with a special focus on aquatic pathogens and suggest some possible avenues for research including the potential to target the T3SS for the development of new anti-virulence drugs.

**Supplementary Information:**

The online version contains supplementary material available at 10.1186/s13567-021-01015-8.

## Introduction

The demand for fish products has been increasing worldwide for multiple decades now, and with the output of fisheries largely stagnant, this demand has been mostly answered through the growth of aquaculture, particularly in developing countries [1]. Aquaculture has become the fastest growing animal food production sector [2, 3] and it is expected to almost double to reach 93.2 million tons in the next decade [4]. This increased production has been in part achieved by increasing the intensity of fish farming, which has led to an increase in the occurrence of infectious diseases, which represent a major limiting factor in aquaculture [5, 6].

Pathogenic bacteria, in particular gram-negative bacteria, are a major group among the pathogens associated with diseases in aquatic organisms and have evolved multiple features to influence their hosts and defend themselves against attackers. Bacteria are separated from their environment by a complex multi-layered envelope. In gram-negative species, this envelope is formed of three layers: the inner-membrane which is composed of a phospholipid bilayer, a thin peptidoglycan cell wall composed of repeating units of N-acetyl glucosamine and N-actyl muramic acid and an outer-membrane, a feature unique to gram-negative bacteria, composed of phospholipids and lipopolysaccharides [7]. The space between the inner and outer-membrane where the cell wall is located is termed the periplasm and can represent a large proportion, up to 40%, of the total volume of the bacterial cells [8]. In addition, many bacteria also secrete a glycocalyx in the form of capsules or slime-layers.

As the main role of this complex envelope is to separate the bacterial cytoplasm from the external environment, it is largely impermeable and movement of non-soluble compounds requires complex transport mechanisms. Notably, virulence factors are by their very definition active outside the bacterial cells and are therefore transferred through specific transport system from the bacterial cytoplasm toward the outer side of the envelope [9]. Several secretion systems have been linked to the export of virulence factors and classified into broad families, forming an ever-expanding list of numbered secretion systems that currently includes at least nine different types [10, 11], although more are very likely to be described in the future. Of course, not all types of secretion systems are present in all bacterial species. For example, the type 7 secretion system is mostly associated with members of the *Mycobacterium* genus and other gram-positive bacteria and no functional members of this family have yet been described in any species of gram-negative bacteria [9, 12].

Among these secretion systems, the best studied, or at least the one associated with the highest number of references in the PubMed database at the time of this writing, is the type 3 secretion system (T3SS). This interest can be explained by the important role of the T3SS in the virulence of many varied bacterial pathogens, including many important pathogens of fish and other aquatic animals.

Because of the importance of the T3SS in the establishment of disease by Gram-negative pathogens, an understanding of their mode of action is necessary for the understanding of many diseases. Moreover, they represent attractive targets in the development of anti-virulence therapy. In aquatic organisms notably, bacterial pathogens have been associated with several outbreaks and much of the bacterial species involved belong to the group of the gram negative, with several known to harbor a T3SS that is often required for the establishment of infections. However, despite this importance, much remains to be learned about the T3SS of many aquatic pathogens. Therefore, the present review was written with the goal to present an overview of what is currently known about the T3SS and list some of the areas still requiring more attention, with the hope that it might help further research on the subject. Since most of the research regarding the T3SS was conducted on pathogens of land animals, the present review will start with an overview of what is generally known about these T3SS, first in term of structure and then regarding their functions and effector proteins. Then, it will focus on what has been discovered specifically about the role of the T3SS in the establishment of diseases in aquatic organisms.

## The T3SS

### Structure of the Type III secretion system

**Figure 1:**
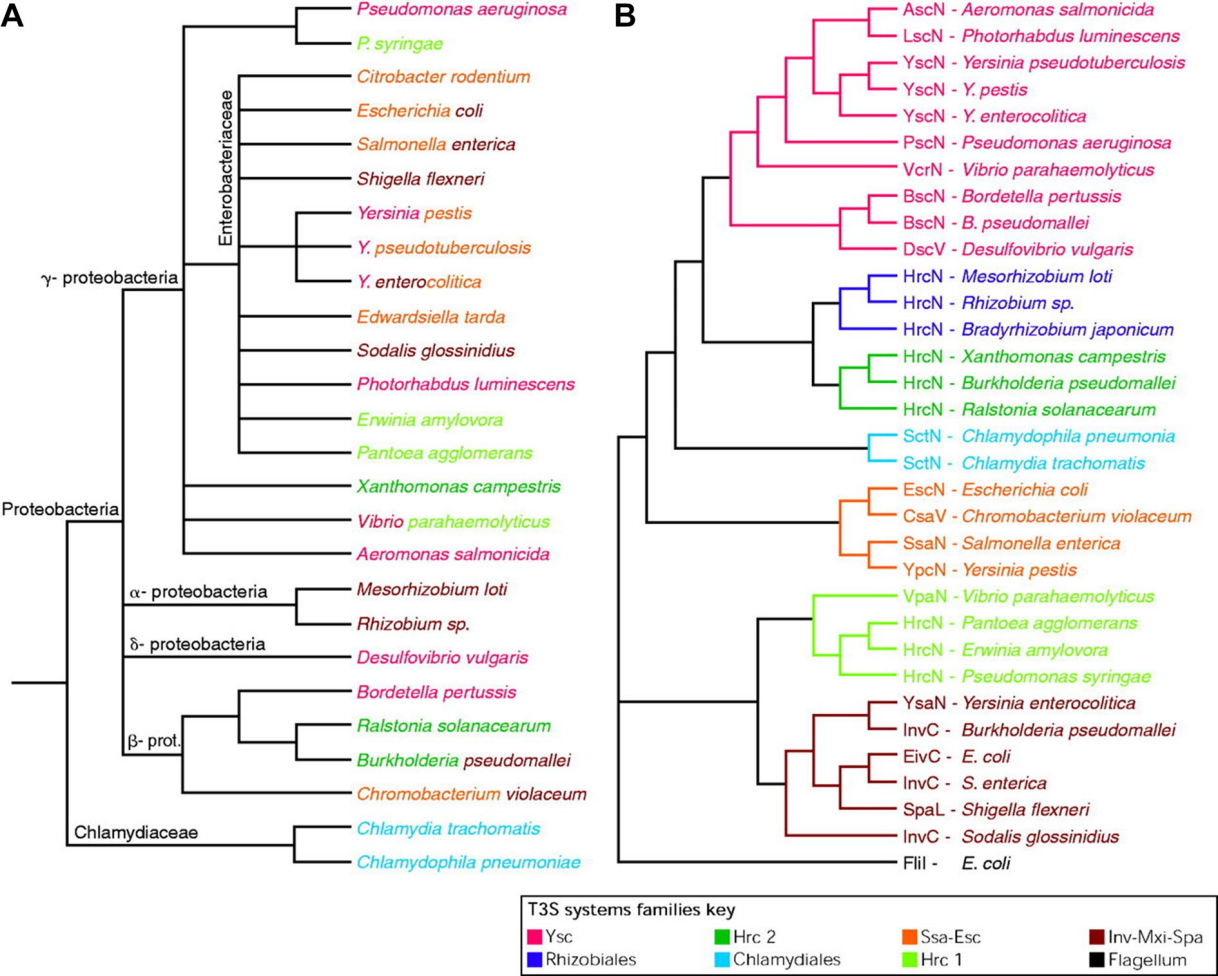
**Phylogenetic tree of the best characterized T3SS families.** The tree was prepared using aligned sequences from the Ribosomal Database Project II and the various sequences were allocated into 7 major families of T3SS based on homology (reproduced with permission from Troisfontaines and Cornelis [16]).

The T3SS either evolved from the bacterial flagellum or shares a direct relative with it, consequently, many structural proteins are homologous between the two apparatus [13–16]. While there are multiple distinct families of T3SS (Figure 1), the Ysc secretion system of the *Yersinia* genus is generally used as the model for our understanding of the T3SS. Moreover, while many species of bacteria share genes homologous to that belonging to the Ysc T3SS, they are often more closely related to T3SS from other genera (Additional file 1). Based on these similarities, seven categories (or families) of T3SS have been defined and most of the T3SS belong to one of these seven families [16]. In recent years, because of the presence of a number of homologous proteins, an effort has been conducted to standardise the names of the various proteins comprising the T3SS following a universal nomenclature based on homology [15, 17, 18]. In this nomenclature, the first 3 letters reflect the T3SS from which the protein originates, while the last letter, written in capital, indicates the specific protein and is identical between homologs. For example, the translocation protein AscE of *Aeromonas hydrophila* is homologous with the translocation proteins YscE in *Yersinia* sp. and SctE encoded by the pathogenicity island 1 (SPI-1) of *Salmonella* [19]. This is the terminology that we will apply in the next paragraphs.

One way to categorise the different proteins composing the T3SS is between conserved and variable proteins. The former is represented by a set of about 15 to 20 conserved proteins [20], mostly playing a structural role, which are homologous between the different organisms harbouring a T3SS [18]. They are generally present in all T3SS and, in a way, define what constitutes this apparatus (Table 1). The second category of proteins is more variable and adapted to the specific bacterium and the role the T3SS play in its lifestyle. Specific effector proteins and corresponding chaperones are therefore most represented in this category.

**Table 1:** **Chaperones effectors corresponding in Enteropathogenic bacteria**

Chaperones	Effectors	Microorganisms	References
SycE	YopE	*Yersinia enterocolitica*	[54]
SycO	YopO	*Yersinia enterocolitica*	[54]
SycT	YopT	*Yersinia enterocolitica*	[52]
SycB	YspB	*Yersinia pestis*	[48]
SycN	YscB	*Yersinia pestis*	[48]
Inv	SopA(SipF) SopE SopE2 SipA(SspA)	*Salmonella*	[182]
SseA SseB SrcA	SseL PipB2 SsaN SteD	*Salmonella enterica*	[44] [55]
CesT	Tir	*Escherichia coli* (EPEC)	[47]
CesT	Map	*Escherichia coli* (EPEC)	[47]
Spa15	IpaA IpgB1, OspC3 OspB	*Shigella flexneri*	[57]
PscE- PscG	PscF	*Pseudomonas aeruginosa*	[40] [59]

**Figure 2:**
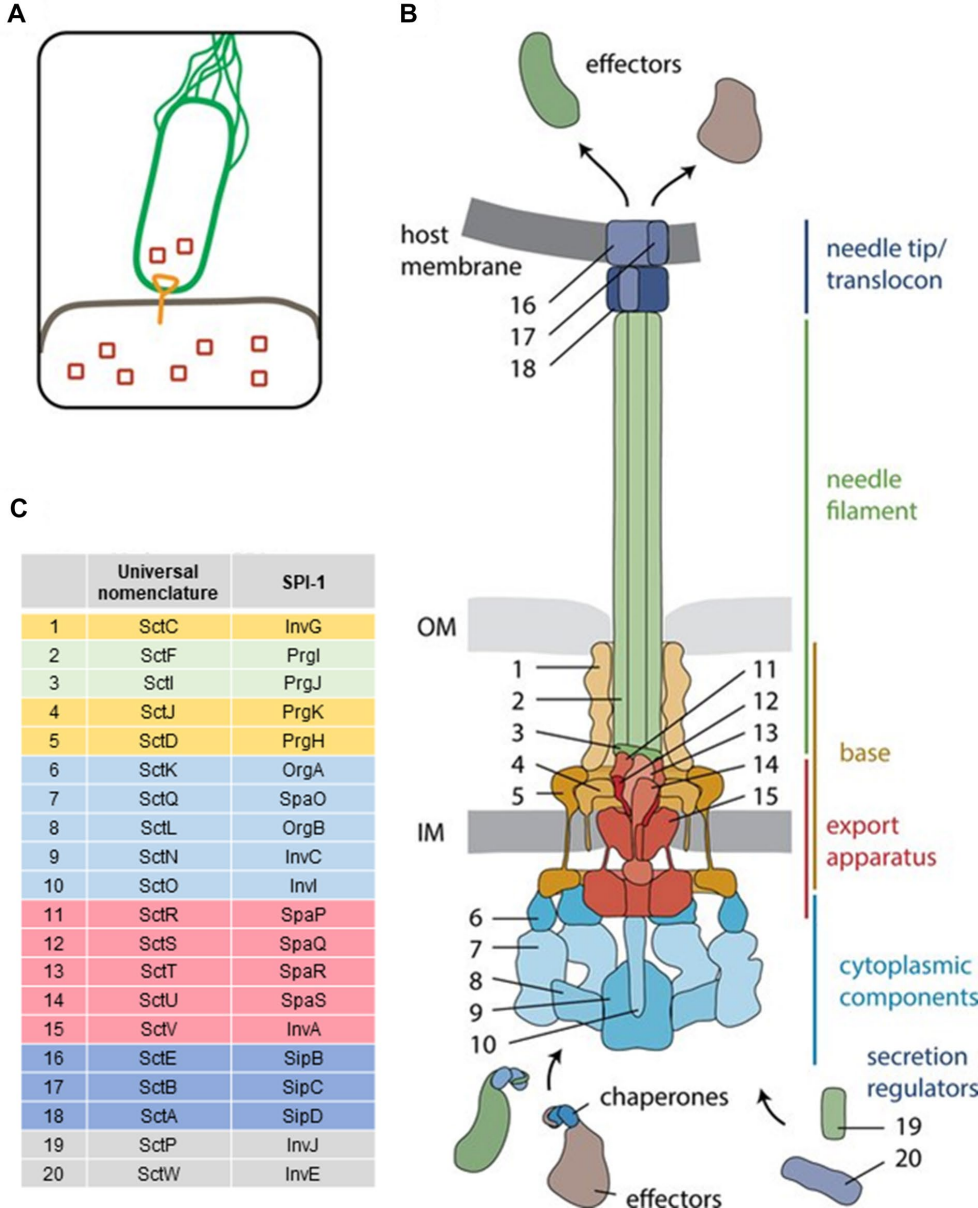
**Schematic of a typical T3SS.** **A** Schematic of the use of the bacterial type 3 secretion system. **B** Common organization and structure of the T3SS. The colors of the proteins correspond to the various areas of the T3SS. **C** Nomenclature of T3SS components displaying both the universal nomenclature and the corresponding protein on the SPI-1 T3SS. Reproduced with permission and minor modifications from Wagner et al. [20].

Another way to characterise this complex apparatus is based on the location of the different proteins. This allows dividing the T3SS into three regions: the cytoplasmic region, the trans-membrane region, also known as the basal body [21], and the extra-cellular region (Figure 2). The main role of the cytoplasmic region is to interact with proteins in the bacterial cytoplasm and to recruit effector proteins before directing them to the rest of the secretory apparatus. Consequently, this section of the T3SS is often characterised as the “sorting platform” or at the “export apparatus” of the T3SS [22]. This region is organised around a polymer formed by the protein YscV which complexes with the proteins YscR, YscS, and YscT [23]. In addition, YscQ, a homolog to the flagellar Motor C-ring is generally considered part of the export apparatus [24]. Energy for the protein export is provided through the ATPase YscN, which forms a hexameric ring and is regulated through YscL [25]. The sorting platform recognises specific substrate to be exported via the T3SS. These substrates are marked by a leader sequence followed by a specific translocation signal, however these are highly variable and ill-defined: unstructured N-terminal sequence appear common in all T3SS substrates but the length of these sequence vary greatly between molecules and some classes of proteins require additional signals to be recognised by the T3SS [26].

The trans-membrane region is composed of two rings forming an export channel through the inner and outer bacterial membranes. The channel in the inner membrane is composed of two proteins and is considered homologous to the flagellar M-ring. YscJ is a lipoprotein, located on the periplasmic side of the inner-membrane and co-purified with YscD [27, 28], which has both cytoplasmic and periplasmic domains [29]. Mutation of the forkhead-associated-domain of YscD was shown to result in silencing of the T3SS in *Yersinia pseudotuberculosis* [29]. YscC belongs to the family of secretin-proteins, it protrudes into the periplasm and forms a pore in the bacterial outer-membrane allowing for protein export into the rest of the T3SS [30]. It is theorised, based on observation performed on T3SS component proteins tagged with fluorescent proteins, that it is the insertion of YsC into the bacterial outer-membrane by the pilotin YscW that initiates assembly of the T3SS, at least as far as the YsC family is concerned, and that the rest of the T3SS is built around YscC [28, 31]. The role of the trans-membrane region is to transport the effector proteins across the bacterial cell membranes. In addition, it is also involved in the secretion of the extra-cellular components of the T3SS on the other side of the cell membranes where they will form the extra-cellular part of the T3SS [32].

Finally, the extra-cellular region can be further separated between the needle that bridges the extra-cellular space between the bacterial and the host cell membrane and the tip and translocon of the T3SS that are inserted into the host cell to act as the injection apparatus and move the effector proteins across the cell membrane. The needle is very conserved and composed of hundreds of repeats of the protein YscF [33]. The tip of the T3SS is formed by the protein LcrV, also known as the V antigen, a polymorphic protein which is absent in many T3SS families and whose function remains unclear [34, 35], although it has been suggested that it plays a role in manipulating the TLR2 mediated immune response. In addition, it is known that YopD/LcrV interactions play a role in the secretion through the T3SS [36] and LcrV are required for the insertion of the other two proteins, YopB and YopD [37]. These two proteins are called translocators and together, they form an integral multimeric 500 to 700 kDa complex referred to as the translocon that acts as a pore into the host membrane, allowing for the delivery of effector proteins directly into the cytoplasm [32, 38, 39]. For this reason, the T3SS is sometime called the “injectisome” [20].

As mentioned above, this description is based on our understanding of the Ysc secretion system, which is likely the best characterised of all T3SS and, while the basic structure is conserved, other T3SS families exist that differs from this model in some respect. For example, the assembly sequences for the basal body and the export apparatus differ between the *Salmonella* Spa and the *Yersinia* YsC secretion systems [40].

### Chaperones of the T3SS

Alongside these structural components, another critical part of the T3SS is the associated chaperones. These chaperones are generally small amphiphilic molecules [41] and are often encoded adjacent to the effector proteins that they serve. They are generally not secreted themselves but may be necessary for secretion and therefore for the T3SS to perform its function [42]. Chaperones can play multiple roles but are mostly known to direct the correct folding of proteins and protect them from untimely degradation, oligomerization and unwanted interactions with proteins in the bacterial membrane [43]. For example, the Inv protein in *Salmonella* directs the folding of cognate secreted proteins in an ATP-dependent manner [42]. A major role of chaperones is to direct their substrate toward the ATPase of the T3SS that will propel them through the T3SS [42, 44]. Chaperones of the T3SS are generally divided within three classes based on their substrate with class I chaperones specialising in the translocation of effector proteins, class II chaperones translocating proteins that assemble to form the secretion pore in the host target membrane, and class III chaperones specialising in the secretion of extracellular filamentous proteins [42, 45].

In enteropathogenic *Escherichia coli*, co-immunoprecipitation assays have shown that the chaperone CesT interacts with both the translocated intimin receptor (Tir) and the Mitochondrial-associated protein (Map). Both gel overlays following migration on SDS-PAGE and enzyme-linked immunosorbent assays have shown that Tir binds to the type III ATPase EscN via CesT [46] which allows for its subsequent secretion through the T3SS [47]. Similarly, crystallography studies have shown that SycN–YscB form a heterodimeric chaperone that permits the secretion of YopN in *Yersinia pestis* [48]. Moreover*,* LcrH plays a role in controlling the levels of secretion of Yop and YopD [49, 50].

An important family of chaperones is the SycE family in the T3SS of members of the *Yersinia* genus [51, 52]. This family includes Syct, a chaperone that harbours a binding site containing residues 52–103 of YopT and is required for the secretion of YopT in *Yersinia enterocolitica* [53]. Three additional chaperones have been described for the Yop of *Y. enterocolitica*, SycE, SycH, and SycO, acting as chaperones for YopE, YopH, and YopO, respectively [54].

In *Salmonella* sp., the multicargo T3SS chaperone SrcA interacts with the ATPase, SPI-2-encoded effector SsaN, and SteD, an adaptor that drives the ubiquitination and degradation of MHCII [55]. Mutation experiments have further shown that SrcA is required for the secretion of both of these effector proteins [44, 55]. Meanwhile, another chaperone, SseA is known to act as a chaperone allowing for the translocation of SseB and SseD [44]. In *Salmonella enterica* serovar *Typhimurium,* mutations in *sseA* has been shown to prevent the assembly of the SPI-2 T3SS and to result in loss of virulence and impaired intracellular replication [56].

*Shigella flexneri* Spa15 chaperone is unusual because it has multiple substrates: it has been shown to enhance the stability of IpaA, IpgB1, OspC3, and OspB [57]. In addition, several homologs of this chaperone have been described, including the SycB that acts as a chaperone for YspB in *Y. enterocolitica* [58].

The PscG–PscF complex in *Pseudomonas aeruginosa* is an example of class III that is required for the assembly of a stable needle for the T3SS. Consequently, mutations in these chaperones have been shown to result in an impairment of the T3SS functions and reduction of its cytotoxic effects on macrophages [59].

### Roles of the T3SS

#### Effects of the T3SS on host cell signalling

Cell physiology is regulated by interconnected networks and intercellular cell signals. A common strategy by microbial pathogens is to interfere with these signals by suppressing or mimicking cellular messengers, notably using effector proteins secreted directly into the cell cytoplasm through the T3SS. Among the common targets of the T3SS are the cytoskeleton, endosomal trafficking, particularly those involved in phagocytosis, mitogen-activated protein (MAP) kinase, and nuclear factor-κB (NF-κB) pathways as well as the innate immunity [60, 61].

The MAP kinase pathway is one of the main cell signals controlling proliferation progression, stress, and inflammation response and plays a vital role in immunity of the host to bacterial pathogens [62]. Two pathways have been described, termed “canonical” and “non-canonical”, that both involve stimulation of membrane receptors of the EGF receptor family, ultimately leading to the cascade activation of transcription factors [62, 63]. Enteropathogenic bacteria in particular interfere with a variety of host cell mechanisms through the mitogen-activated protein kinase (MAPK) and NF-κB pathways [61]. In *Yersinia*, screening of host genes using RNA interference allowed to identify 19 NF-κB-regulated genes [64]. These genes included the heat shock protein H 1 (HSPH1) and encompass a variety of functions, including the NF-κB, the MAPK and the ERK signalling pathways, ion channel activity, and even cell growth regulation. Normally, these genes are upregulated by NF-κB, although this does not occur in the presence of wild-type *Y. enterocolitica*. However, curing this bacterium from the virulence plasmid pYV (that carries the bacterium T3SS) restores expression of these genes in infected cells, leading the authors to conclude that they were the target of *Y. enterocolitica* T3SS [64].

Moreover, research on the T3SS effector NleB1 from enteropathogenic *E. coli* showed that the effector NleB1 binds to the death domain of the Fas-associated protein [65]. This binding prevents the formation of the canonical death-inducing signalling complex (DISC) and the proteolytic activation of caspase-8 in order to avert apoptosis [65]. Similarly, another T3SS protein has been described as preventing cleavage of caspase-8, caspase-3, and the receptor-interacting serine/threonine protein kinase 1 (RIPK1) as well as binding caspase-4, -8, and -9 to inhibit their activity [66].

#### Cytotoxic effects

The cellular cytoskeleton is a network of actin and microtubules that act together to give shape and rigidity to eukaryotic cells while also playing a role in cellular trafficking, cellular uptake and cell movement. A major aspect of the cytoskeleton is the Rho family that constitutes a subfamily of the broader Ras super-family. Rho are small GTPases modulating actin organization, cell cycle progression and gene expression and playing a role in the organization of guanine exchange factors (GEF) and GTPase activating proteins (GAP) to change the inactive GDP- bound to active GTP- bound [67].

Members of the Rho family are a major target for effector proteins of the T3SS, either for inhibition or activation of these networks. For example, in *Yersinia* spp., translocation of the recruiting effector YopD through the cell membrane allows for the formation of channels through which YopE is delivered into the cell cytoplasm [32]. YopE GTPase inactivates members of the Rho family, including RhoA, Rac1, but not Cdc42 which is not considered an in vivo target for YopE [68]. This inactivation leads to disruption of the actin cytoskeleton [68, 69] and elicits a proinflammatory signalling response culminating in cytotoxicity [70]. However YopE is also recognised as a danger signal by macrophages and it has been shown that presence of YopE increased macrophage mediated killing of *Yersinia* bacteria [71]. Another effector of *Yersinia* spp., YopT, is a cysteine protease that induces apoptosis of macrophages through the inhibition of the MAPK and NFκB pathways [72], disturbing the F-actin structure of the cytoskeleton and resulting in a rounding of affected cells [73]. Interestingly, YopT competes with YopE for the same pool of Rac1, and the two effector proteins have antagonistic properties [71]. Similarly, YopK/YopQ (the name varies between homolog in different species of *Yersinia*) plays a role in the control of Yop translocation across the host cells [74]. In *Y. pseudotuberculosis*, YopQ has been associated with cytotoxic effects on polymorphonuclear leucocytes, although the precise mechanisms of actions remain unclear [75]. Similarly, YopJ effector has an inhibitory function for MAPK and NF-κB [76]. This effector prevents phosphorylation of serine and threonine residues MAPKK6 and interrupts the pathway [76, 77], reducing the production of the anti-apoptotic regulator Bcl-2 and leading to apoptosis of infected macrophages [78]. Moreover, YopE interferes on the signalling by acetylation of MEK2, MEK6 and, IKK as well as promoted apoptosis in infected macrophages [72, 78].

*Salmonella enterica* also induces apoptosis in macrophages through an unknown NF-κB-independent mechanism that relies on signalling through the kepro-inflammatory activator caspase 1 [61, 63, 79, 80]. Ectopic expression of the SPI-2 T3SS effector, SseL, did not support a role in the down-regulation of the host immune response and in particular the NF-kB pathway [81].

#### Other means of circumventing the immune response

In addition to causing apoptosis, bacterial pathogens can protect themselves from phagocytosis and the immune system by interfering with the host cells in other ways. For example, YopE and YopT can hinder the maturation of prointerleukin-1β in macrophages [82]. Meanwhile, EspF and EspJ of *E. coli* inhibit the PI3K and Src signalling required for phagocytosis [61].

Moreover, in *Citrobacter rodentium* and other Attaching/Effacing (A/E) pathogens like *E. coli*, the metalloprotease effector NleC cleaves the transcription factor p65, a transcription factor involved in the transcription of NF-κB in colon epithelial cells isolated from the intestine of mice. The resulting p651–38 fragment then interferes with p65/ RPS3 interactions to hinder the transcription of NF-κB and NF-κB signalling [83]. Likewise, the effector SpvB secreted by the Salmonella pathogenicity island-2 (SPI-2) T3SS of *Salmonella enterica* serovar Typhimurium has been shown to promote the expression of the E3 ligase KEAP1 [84]. KEAP1 acts as a repressor for IKKb which is in turn a promoter of NF-kB, therefore this effector proteins allows the bacterium to supress the NF-kB-dependent signalling pathway associated with the immune response [84].

Two distinct T3SS have been described in members of the *Salmonella* genus, located on the SPI-1 and SPI-2 and correspondingly named T3SS1 and T3SS2 [85, 86]. Both of these T3SS are required for bacterial virulence: one of the pathways of intracellular infection in *Salmonella* spp. involves the formation of salmonella containing vacuoles (SCV) in which the bacterium can multiply overtime [87]. *Salmonella* spp. requires both T3SS1 and T3SS2 to survive within these SCV and effector proteins interfere with the maturation of the vacuoles [87], although the precise mechanisms involved remain unclear. Effectors from T3SS1 are generally detected earlier in the infection process and it has been suggested that they are more involved with the earlier stages of the vacuole maturation while effectors from T3SS2 are more involved with later stages [87]. Finally, experiments using mutants of *Acidovorax avenae* identified an effector protein (*A. avenae* K1 suppression factor 1) that was associated with supressing the cellular response following recognition of flagellin associated molecular patterns [88].

#### Interference with the cytoskeleton to promote internalisation and cell invasion

Finally, a major role of the T3SS is in tissue colonisation to assist with bacterial attachment and to provide a mechanism of intracellular invasion through the trigger mechanism. Tir molecules are effectors which exist in two distinct forms, secreted by enteropathogenic and enterohaemmorhagic *E. coli*, respectively [89]. Both forms of Tir stimulate actin assembly through Nck-independent pathways leading to the recruitment of F-actin at the site of contact and the formation of pedestal structures to which the bacteria can then attach tightly [89].

A large class of effector proteins, best known in *Shigella* spp. and *Salmonella* spp., are characterised by their Trp-X-X-X-Glu motif [90]. These proteins are known to activate the Rho GTPases RhoA, Cdc42, and Rac1, resulting in the reconfiguration of the cytoskeleton and the formation of stress fibres, filopodia, and ruffling of the host cell membrane, leading to the intracellular uptake of the bacteria [90–92]. Similarly, the effector proteins SopE, SopE2, and SigD of *Salmonella* can activate actin in a Arp2/3-dependent manner [93], resulting in membrane ruffling that facilitates bacterial invasion [94]. Following internalisation, the activation of Cdc42 and Rac is reversed by SptP, another effector protein while IpgD destabilizes tight junctions, facilitating the formation of SVC [93–95].

## T3SS in aquatic pathogens

As mentioned, many T3SS-harbouring bacterial pathogens have been associated with diseases in fish and other aquatic animals. However, despite this importance, much remains to be learned about these virulence factors. A review of what is currently known is presented in the following pages.

### *Edwardsiella* spp*.*

The *Edwardsiella* genus represents a small but growing group of facultative anaerobic enteric bacteria and includes pathogens causing disorders in both fish and mammals. Mutation studies performed on 7 genes from the T3SS of *E. tarda* resulted in an increase in the bacterium LD_50_ (at least tenfold) as well as a decreased survival and growth of the bacterium in fish phagocytes following silencing of the T3SS [96]. The first effector of T3SS discovered in *E. tarda* was EseG whose secretion is dependent on the activity of the EscB chaperone [97]. EseG shares partial homology with the effectors SseG and SseF of *Salmonella* spp. and like these two proteins was found to interact with the cytoskeletons of the host cells, suggesting that it too might be involved in promoting internalisation of bacteria into fish cells [97]. In addition, the inner rod protein of the T3SS, alongside the flagellin molecule, induces pyroptosis activating the death process in host cells [98]. Pyroptosis starts with the activation of caspase-1 via the inflammasome complex, resulting in the secretion of IL-1β, IL-18 and TNF-α and concluding in cell death [98]. The protein EsaE is a membrane bound protein required for the secretion of T3SS effectors and its deletion has been shown to result in the attenuation of the bacterium [99]. Liu et al., furthermore demonstrated that the expression of *E. tarda* T3SS was regulated through a ternary complex involving EsaB, Esa, and EsaM [100]. Notably, they found that the activity of EsaL was pH dependent with EsaL suppressing secretion at pH 7.2 but promoting it under acidic conditions (pH 5.5) [100].

*E. ictaluri* is best known as a pathogen of catfish and sequencing of *E. ictaluri* plasmids pEI1 and pEI2, revealed remarkable similarities with the T3SS of *E. tarda* as well as SlrP, SspH1, and SspH2 in *Salmonella typhimurium* and IpaH of *S. flexneri*. pEI2 has also limited similarities to Spa15 of *S. flexneri* 5 and InvB of *Shigella sonnei* and *S. typhimurium* [101]. Signature-tagged mutagenesis have shown that these regions were necessary for the ability of *E. ictaluri* to cause infections [102, 103]. Both acidification and subsequent neutralization of the *Edwardsiella* containing vacuoles (ECV) are required to trigger the translocation of the *E. ictaluri* effectors [103], a feature that appears unique among bacterial pathogens. In contrast, transcription and assembly of the related *Salmonella* SPI-2 T3SS only requires acidification of the SCV [104]. The EseN effector plays an important role in *E. ictaluri* invasion, where it activates extracellular replication in catfish head kidney by dephosphorylation in vivo in the head kidney of infected fish, and ex vivo in macrophages derived from the head kidney [105]. EseN can also shut down signal-regulated kinases 1 and 2 (ERK1/2) early in the infectious process [80, 105]. Furthermore, there is evidence of reduced numbers of bacteria in infected tissue and in mortality in channel catfish (*Ictalurus punctatus*) infected with bacteria with a deleted eseN gene bacteria compared to wild-type infected tissue [105]. Finally, low pH and low phosphate triggers the expression of the T3SS effectors EseB, EseC, and EseD expression as well as the PEI1and PEI2 encoded, ESeH and ESeI [106].

*Edwardsiella piscicida*, has been described more recently and has been associated with diseases in aquatic animals, e.g. catfish and zebrafish [107]. It possesses both a T3SS and a T6SS, which are responsible to transport 12 effectors with synergistic activity [108]. In-frame deletion mutants of the T3SS structural genes EpDssaV, EpDesaM, EpDyscR in *E. piscicida* have resulted in loss of virulence in the bacterium and further analysis found that the mutants had potential efficacy as vaccines in catfish fingerlings [109]. The T3SS *E. piscicida* triggers TNF-α and IL-1β, synchronously, and activates inflammatory cytokines contributing to spreading the infection in host cells [110]. More recently, transcriptomic analysis using the regulator EsrB have allowed to identify 6 novel effector proteins that were co-expressed alongside proteins of the T3SS, although the exact role of these effectors remains to be elucidated [111].

### *Vibrio* spp.

*Vibrio* species include a wide array of pathogenic bacteria found in both marine and terrestrial animals and these bacteria are considered endemic in water reservoirs throughout the world and are uncommon in the fact that many members of this genus commonly harbour two chromosomes, of varying size [112]. Most members of the genus *Vibrio* only harbour one T3SS [113]. However, sequencing has shown that several pathogenic strains of *Vibrio parahaemolyticus* harbour two distinct T3SS gene clusters encoding two different apparatus known as T3SS1, homolog to the one shared by other *Vibrio* spp., and T3SS2, carried on the main and secondary chromosome, respectively [113].

The T3SS1 showed an organisation comparable to that of the T3SS of *Yersinia* spp. [114]. Mutant strains of *V. parahaemolyticus* deficient in either T3SS1 or T3SS2 activity have been generated by Park et al. [114] and infection studies using these mutants have shown that T3SS1 was involved in the cytotoxic activity of the bacterium [114]. Activity of the T3SS1 on fibroblasts has shown an impact on the expression of 398 host genes [115]. Multiple T3SS1 effector genes have been identified [113], these include the effectors VopQ, VopS, VPA0450 and VopR (VP1683). VopQ has been linked to cell autophagy and toxicity via JNK-pathways leading to the expression of IL-8, caspase-1, IL-1β, and IL-18 and the inactivation of Cdc42, culminating in cellular cell death [116–118]. Moreover, the fic domain of VopS promotes cytoskeleton destruction via RhoA, Rac1 and Cdc42 (Rho GTPases) AMPylation [116, 119]. VopS and VopQ are homologous proteins, collaborating to promote cell autophagy concomitantly [119–121]. Moreover, VPA0450 was reported to disrupt the bacterial cell membrane by interfering with the cytoskeleton, resulting in membrane blebbing [122]. In addition, the regulators EsxA, EsxC, ExsD and ExsE have been shown to be necessary for the expression of the T3SS1. ExsA acts as a positive transcriptional regulator and plays a regulatory role for T3SS1 and shows DNA binding motifs linked to multiple T3SS1 genetic operons and promotes the expression of the T3SS1. ExsD binds ExsA to block expression of the T3SS1 genes while Exsc binds ExsD to allow for their expression [123]. Expression of the T3SS is regulated through the regulatory protein ExsA and several environmental factors like temperature above 30 °C or elevated presence of magnesium in the medium have been correlated with an increased activity [124]. T3SS1 is further involved in suppressing inflammatory responses through the inactivation of Cdc42 which otherwise acts as a stimulator to activate inflammasomes, NLRP3 and NLRC4 in host cells [117]. In *V. alginolyticus*, the effector proteins Val1686 and Val1680 include a Fic-domain, and have been shown to induce apoptosis, cell rounding and cell lysis through Val1686-dependent Rho GTPase inhibition [125]. However blocking the caspase 3 apoptotic pathway does not prevent cell rounding, membrane pore forming or cell death, suggesting that this T3SS can act through other cellular pathways beside caspase 3 [126]. Conversely, the T3SS1 was also shown to upregulate several pathways involved in cell-survival, a feature that the authors suggested might help the bacterium reduce the immune response by masking its cytopathic activity [115].

Somewhat less information is available regarding the rarer T3SS2. Screening of 155 environmental isolates (defined by the absence of the thermostable direct haemolysin (TDH) and TDH-related haemolysin) for genes coding for 2 elements of the T3SS2 (*vscC2* and *vopP*), detected these genes in 10 or 8 isolates, respectively [127]. This suggested that this virulence factor might be uncommon in environmental isolates. Similarly, screening of environmental isolates from grouper and milkfish suggested that presence of T3SS2-associated genes was also variable, ranging from 14 to 100% of the isolates, depending on the gene [128]. When present, the T3SS2 has been linked to enterotoxicity [129, 130]. Notably, the T3SS2 is necessary for the secretion of the effector protein VopT which is a homolog of the *Pseudomonas* exoenzyme T (ExoT) [130]. Expression of VopT has been shown to inhibit growth in yeast culture while mutant of *V. parahaemolyticus* deficient in VopT displayed reduced cytotoxic activity against Caco-2 cells [130]. However, compared to the T3SS1, the T3SS2 appears to only be active against a limited number of cell lines [131]. Finally, Calder et al. have described the role of the T3SS2 in the formation of biofilms [132].

### *Aeromonas* spp.

*Aeromonas salmonicida* is another major pathogen in aquaculture and the causative agent of furunculosis [132, 133]. The T3SS of *A. salmonicida* is likely its most important virulence factor [134] but was only relatively recently reported, its discovery probably hindered by the fact that T3SS are lost when the bacterium is cultivated above 20 °C [135–137], due to the T3SS gene cluster being locating in a thermosensitive region [138]. Among the most important effector proteins secreted by the T3SS of *A. salmonicida* are AopH, Ati2, AexT, AopP, AopO, AopN and ExsE [134], that have been associated with cytoskeletal collapse and immune system response impairment [139], with *aopO* being upregulated during the infectious process [140]. Moreover, AcrV and AopB are homologues in LcrV and YopB in *Yersinia* which stimulate IL-10 production in the cell, consequently supressing the immune response [141].

More recently, experimental infections of *Oncorhynchus mykiss* by wild-type *A. salmonicida* harbouring a fully functional T3SS, a mutated T3SS ascV (ΔascV), or a strain in which the T3SS had been completely lost confirmed the central role of the T3SS in the establishment of disease, with ΔascV being attenuated and the T3SS-less strain being totally avirulent [142]. Moreover, the authors also performed RT-qPCR on the anterior kidneys of infected fish showing a down-regulation of several immune genes associated with T3SS activity. Notably, expression of the interleukin 2 (a cytokine regulating proliferation of T-cells) and interferon gamma (a cytokine produced by T-cells) as well as that of the markers CD4 and CD8 (both expressed by different sub-populations of T-cells) were very strongly downregulated in fish infected with the wild-type and ΔascV strains but not in the strain without T3SS [142]. These results suggested that the T3SS of *A. salmonicida* had a strong immunosuppressive effect, particularly targeting different populations of T-cells, even if the precise effectors involved remain to be elucidated [142].

The effector AexT has been linked to cell cytotoxicity. It is considered homologous to *P. aeruginosa* bifunctional toxins exoenzyme S (ExoS) and exoenzyme T (ExoT), having a GAP function, activating members of the Rho family and resulting in the depolymerisation of ADP-ribosyltransferase actin and cell rounding [143]. Studies in mice have shown that deletion of *aexU* gene from the genome of *A. salmonicida* resulted in loss of virulence [144]. Furthermore, immunization of mice with recombinant AexU protected them from subsequent lethal challenge dose by the wild-type bacteria.

Notably, presence of T3SS genes is less systematic in *A. hydrophila*, the other major fish pathogen in this genus [133], as only some of the strains of the bacterium harbour all the genes for a functional T3SS [145]. However, sequencing of the gene cluster of *A. hydrophila* AH-3 using primers derived from sequences in *A. salmonicida* revealed the presence of 35 T3SS genes, 20 of these genes were homologous to genes on *A. salmonicida* and at least half of the remaining 15 proteins appeared novel to *Aeromonas* [19]. Silencing of the T3SS by deletion of the *ascV* gene resulted in loss of virulence in both rainbow trout and mice, showing the role of the T3SS in the disease process [19]. This decreased virulence of the mutants was confirmed in dwarf gourami (*Trichogaster lalius*) and was associated with reduced cytotoxicity and increased phagocytosis, which could highlight some mechanisms of action of this T3SS [146]. Epidemiologically, screening for the presence of several genes associated with the T3SS showed that the strains harbouring such genes were more likely to be associated with outbreaks of disease [145]. Similarly, screening of *Aeromonas* spp. from human patients has also shown that *Aeromonas caviae*, generally regarded as less virulent, were less likely to harbour a T3SS than other members of the genus [147]. The regulatory mechanisms of this T3SS in *A. hydrophila* have been investigated and it was shown that it was expressed in response to several environmental factors, including calcium depletion, high magnesium concentration, and high temperature [148]. The latter makes an interesting contrast with *A. salmonicida* where the T3SS is lost at high temperature. As previously stated, several of the effector proteins secreted by the T3SS of *A. hydrophila* are homologous or present similarities with those of *A. salmonicida.* For example, the first part of the *aexT* gene is identical in *A. hydrophila* and *A. salmonicida* [149], while the second half is different. The same study identified the protein ADP-ribosyltransferase activity and showed that mutation of *aexT* resulted in a slight reduction of their virulence both in vitro and in vivo in both three-spot gourami (*Trichogaster trichopterus*) or mice [149]. Similarly, *aexU* was found to have similarities with *aexT* of *A. salmonicida* [144, 150]. In addition, this protein also demonstrated GAP activity resulting in the disruption of actin filaments, inhibiting cytokine secretion and resulting in apoptosis in HeLa cells [151]. Like for *A. salmonicida*, deletion of the gene resulted in increased phagocytosis and decreased virulence of the bacterium [144, 150, 151]. Despite these advances, much is still to be learned about the T3SS of *A. hydrophila*. Notably, the precise targets and mechanisms of actions of the known T3SS effectors remain to be determined. Moreover, because of the variability in T3SS between isolates and the limited numbers of strains that have been investigated, it is likely that a significant number of effector proteins remains to be identified.

Finally, Matys et al*.* have recently reviewed the effector proteins secreted by bacteria of the *Aeromonas* genus, and this article should be of great interest to readers [152].

### *Flavobacterium* spp.

The genus *Flavobacterium* encompasses many species and is ubiquitous in aquatic and soil environments. Among these species, several are well known as opportunistic or true pathogens. In particular *Flavobacterium psychrophilum* which is a major problem in particular in fish hatcheries, and *Flavobacterium columnare*, which is mainly known as a pathogen in warm water fish, notably in *I. punctatus* [153]. Even within a species, it is known that different strains can vary wildly in term of their virulence [154, 155]. For example, in *F. columnare*, examination of the bacterial genome has allowed to subdivide members of the species in several subgroups termed genomovars [156, 157], and these genetic groups appear to have at least some correlation with the virulence of the isolates [155]. T3SS are not unknown within this genus: examination of the genomes from two strains belonging to two different genomovar revealed the presence of a partial T3SS on the genome of *F. columnare ATCC* 49512, considered avirulent in catfish, and a complete T3SS on the genome of *F. columnare* 94-081, belonging to the genomovar II and considered highly virulent [158]. Similar examination performed on 4 strains belonging to genomovar I with varying degrees of virulence identified T3SS on all genomes [159] and suggested that differences in virulence were likely related to differences in chemotaxis and bacterial adhesion rather than the T3SS. Overall, the T3SS of *Flavobacterium* spp. does not appear to have garnered much attention. In the future, it might be of interest to systematically investigate the prevalence of this virulence factor within both clinical and environmental isolates of *Flavobacterium* spp. as well as clarify the role that it might play in the establishment of disease by deletion mutation or other silencing of the genes involved.

### *Yersinia ruckeri*

*Yersinia ruckeri* is a major pathogen, particularly well-known in salmonid fish, and associated with generalized bacteraemia and septicaemia [160]. The presence of a T3SS in *Y. ruckeri* was reported by Gunasena et al. in 2003 [161]. Subsequent sequencing of the complete *Yersinia ruckeri* SC09 genome has allowed to further confirm the components of a ysa T3SS [162]. This T3SS is unusual because of its chromosomal location and it is carried on an operon with a moderate (ranging from 60 to 37%) identity to four genes of *Y. enterocolitica* [161]: YsaV (protein ID: CNI46870.1), YsaK (protein ID: CNI46839.1), YsaN (protein ID: CNI46802.1) and CDS19 (protein ID: NZ_KN150747.1). Furthermore, it displays similarities in gene sequence, arrangement and gene content with that of *Y. enterocolitica* biotype 1B and SPI-1 [163]. However, beside the sequencing of these genes, no information is currently available regarding the function of this T3SS and of their role in the virulence, internalization, replication and invasion mechanisms [162, 164]. This is particularly surprising considering the importance of *Y. ruckeri* as a fish pathogen and the amount of research that have otherwise focused on this pathogen.

### *Pseudomonas* spp.

*Pseudomonas* spp. are gram-negative bacteria belonging to the γ-proteobacteria [165] that can display a high level of resistance for antibiotics [166] and infect a wide range of animals including human and fish [167, 168]. For example, *P. aeruginosa* is a normal part of the fish microbiota but may behave as an opportunistic pathogen in immunocompromised fish, resulting in ulcers and hemorrhagic septicemia [168]. The T3SS of *P. aeruginosa* is similar in structure with that of other gram-negative bacteria like *Salmonella, Shigella* and *Yersinia* spp. [169] and it has been theorized that this T3SS might have evolved to kill environmental amoeba as a defense mechanism against predation [170]. Four effector proteins (ExoT, ExoS, ExoU and ExoY) have been described in *P. aeruginosa*. Intriguingly, ExoS and ExoU are, for unknown reason, rarely secreted together in the same strain and have been associated with apoptosis and rapid cell lysis, respectively [166]. Furthermore, deletion mutations have shown that ExoU plays a role in damaging the lung epithelium in mice [171]. The other two effector proteins include ExoT and ExoY. ExoT has been linked to apoptosis in cultured cells following disruption of the mitochondrial membrane and leaking of cytochrome C into the cytosol [172, 173]. Furthermore, this effector also interferes with the actin cytoskeleton in order to inhibit phagocytosis, as well as slow wound healing by preventing epithelial cell migration in order to facilitate bacterial colonization [174]. The last known effector protein is ExoY which promotes secretion of the cyclic pyrimidine nucleotides cGMP and cUMP which play an important regulatory role in apoptosis and ion channel regulation, as well as smooth muscle control, including in the blood vessels [175].

While most of this work has been conducted on mammalian models, and in particular a mouse model, the proteins targeted by *P. aeruginosa* are well conserved and it is plausible that the T3SS has similar effects on fish cells. Moreover, these mechanisms of action, notably induction of apoptosis, escape from the immune system, and epithelial damage and delayed healing are consistent with the clinical signs associated with *P. aeruginosa* infections in fish [168].

## New potential avenues for research

While T3SS appear commonplace among aquatic bacterial pathogens, they are not universal in every species. Therefore, it would be of interest to systematically screen and sequence both clinical and environmental bacterial isolates to identify the presence of T3SS. This is particularly the case for members of the genus *Aeromonas* and *Flavobacterium* in which the presence of T3SS is only sporadic. Moreover, comparing the prevalence of these virulence factors between clinical and environmental isolates would provide us with some understanding of their role in disease.

In addition, sequencing of the T3SS regions would allow to identify some of the effector proteins based on homology with previously characterized proteins in other species. This approach could be complemented by mutation experiments targeting structural element of the secretion apparatus followed by a comparison of the mutant secretome to that of the wild type isolate. Effector proteins are the most variable part of the T3SS, including between isolates of the same species, therefore there are likely many such proteins that remain to be identified and this approach has the advantage not to be reliant on sequence homology with already known effector proteins.

Moreover, the mutants could then be tested for infection and virulence, both in vivo and in vitro, to assess their role in the infection process. This approach would be relevant for bacterial fish pathogen in which the presence of a T3SS is known but its role in the establishment of disease remains unclear, like in *Y. ruckeri* or *Flavobacterium* spp. The specific mechanisms through which the expression of these T3SS is regulated is also an area of interest as is the target and mechanisms of actions of individual effector proteins. The later could be investigated by transfecting cells with plasmids expressing the gene of interest. The effect of this transfection on the cells physiology could then be investigated [176]. Molecules belonging to the T3SS would also represent interesting targets for the development of prophylaxis or therapeutic treatments. For example, the T3SS of *P. aeruginosa* has been targeted and several small molecules have been identified with inhibitory properties on the T3SS [166] as well as antibodies targeting the protein PcrV [177]. Similarly, additional vaccine attempts have been conducted targeting the homologous LcrV on *Y. pestis* [177] and AopO, a T3SS effector of *A. salmonicida*, has proven immunogenic in *O. mykiss* [140]. Targeting virulence mechanisms rather than the pathogen itself is advantageous not only because it offers new targets but also because it is generally accepted that it exerts a lower selective pressure on the pathogen, leading to a delayed emergence of resistance mechanisms [178]. Currently, most of these research efforts are being conducted on human pathogens; however, it is highly plausible that many of these therapeutants will be equally effective on fish pathogens. Finally, interference with the T3SS could also be performed by targeting its regulatory mechanisms. In particular, some of T3SS are known to be regulated through quorum sensing and strategies that target quorum sensing molecules, such as quorum quenching probiotics, have already been identified in fish health management [179, 180].

## Conclusions

T3SS constitute a way for bacterial pathogens to manipulate the physiology of the host cells. They represent a powerful and versatile tool, and some of the most important virulence factors of gram-negative bacteria. Several examples show that it is also the case for multiple bacterial fish pathogens. However, comparatively less is known about their exact repertoire of secreted effector proteins as well as the targets and precise mode of action of these effectors. This is for example the case of otherwise well-known pathogens such as *Y. ruckeri* or members of the genus *Vibrio* or *Aeromonas*. In this context, much research effort, as detailed above, is still required to improve our understanding of the role of the T3SS in aquatic bacterial pathogens, how common they are and how they contribute to specific diseases as well as clarifying the factors governing their expression. Moreover, molecules belonging to these T3SS might also represent new targets for the development of vaccines or even new therapeutic treatments.

## Supplementary Information

**Additional file 1.** Proteins belonging to the seven main T3SS families classified based on their homology. The table is reproduced with permission from Gazi et al. [181].

## Data Availability

The data supporting the conclusions of this article is included within the article.

## Abbreviations

A/E pathogens: Attaching/effacing pathogens
ATPase: Adenosine triphosphatase
Bp: Base pair
CD4: Cluster of differentiation 4
CD8: Cluster of differentiation 8
Cdc42: Cell division control protein 42 homolog
DISC: Death-inducing signalling complex
cGMP: Cyclic guanosine monophosphate
cUMP: Cyclic uridine monophosphate
ECV: *Edwardsiella* Containing vacuoles
ERK: Extracellular signal-regulated kinases
ExoS: Exoenzyme S
ExoT: Exoenzyme T
Fic domain: Filamentation induced by c-AMP domain
GAP: GTPase- activating protein
GEF: Guanine exchange factors
GTPase: Nucleotide guanosine triphosphatase
HSPH1: Heat Shock Protein H 1
IL-1β: Interleukin 1 β
IL-2: Interleukin 2
IL-8: Interleukin 8
IL-10: Interleukin 10
IL-18: Interleukin 18
IKKb: Inhibitor of Nuclear Factor Kappa B Kinase Subunit Beta
JNK pathway: C-Jun N-terminal kinase pathway
kDa: Kilodalton
Keap1: Kelch ECH associating protein 1
Map: Mitochondrial associated protein
MAPK: Mitogen-activated protein kinases
M ring: Membrane ring
NF-κB: Nuclear factor-κB
RAC1: Ras-related C3 botulinum toxin substrate 1
RAS: Rat sarcoma viral oncogene homolog
RIPK1: Receptor-interacting serine/threonine protein kinase 1
Rho: Ras homolog
SCV: Salmonella containing vacuoles
TDH: Thermostable direct haemolysin
Tir: Translocated intimin receptor
TLR: Toll-like receptor
TNF-α: Tumour necrosis factor α
T3SS: Type III secretion system
Yop: Yersinia outer protein
Ysc: Yop secretion
